# Location of Host and Host Habitat by Fruit Fly Parasitoids

**DOI:** 10.3390/insects3041220

**Published:** 2012-11-22

**Authors:** Serge Quilici, Pascal Rousse

**Affiliations:** CIRAD (Centre de Coopération Internationale en Recherche Agronomique pour le Développement), UMR PVBMT Cirad, Université de La Réunion, Pôle de Protection des Plantes, 97410, Saint-Pierre, France; E-Mail: rousse.pascal@wanadoo.fr

**Keywords:** host habitat, host location, tephritid parasitoids, chemical stimuli, visual stimuli, mechanical stimuli

## Abstract

Augmentative releases of parasitoids may be a useful tool for the area-wide management of tephritid pests. The latter are parasitized by many wasp species, though only a few of them are relevant for augmentative biocontrol purposes. To date, nearly all the actual or potential biocontrol agents for such programs are egg or larval Opiinae parasitoids (Hymenoptera: Braconidae). Here, we review the literature published on their habitat and host location behavior, as well as the factors that modulate this behavior, which is assumed to be sequential; parasitoids forage first for the host habitat and then for the host itself. Parasitoids rely on chemical, visual, and mechanical stimuli, often strongly related to their ecology. Behavioral modulation factors include biotic and abiotic factors including learning, climatic conditions and physiological state of the insect. Finally, conclusions and perspectives for future research are briefly highlighted. A detailed knowledge of this behavior may be very useful for selecting the release sites for both inundative/augmentative releases of mass-reared parasitoids and inoculative releases for classical biocontrol.

## 1. Introduction

The location of the habitat (including the micro-habitat) and host by female parasitoids is a theoretically sequential behavior leading a parasitoid to a potential host, which is then examined for its suitability (*i.e.*, the host acceptation phase) [1]. During the location process, numerous studies have shown that female parasitoids respond to various stimuli from the plant, the host population, the host itself or their interactions: those stimuli are mainly volatile semiochemicals, though visual and/or mechanical cues are also used [1,2,3]. [4] showed that the stimuli which are reliably associated with the presence of hosts are preferably used. Volatile semiochemicals have been by far the ones most studied. They have been classified into kairomones, allomones or synomones according to cost/benefit considerations [5].

The location of both the host habitat and host is a plastic and flexible behavior. In addition to the genetic background and the physiological state of the parasitoid, the experience acquired by the female is an important factor inducing variability in foraging behavior of many parasitoid wasps, after [4,6,7] or even prior [8] to the emergence of the adult parasitoid. A common rule states that the more generalist the parasitoid is, the more it relies on experience to locate its host [9] but this assumption needs to be amended by additional considerations on optimal foraging, such as the variability of the environment [4] or the life history traits of the parasitoid [10,11].

Knowledge of host foraging behavior of parasitoid wasps is of considerable importance for the effective use of parasitoids in pest management, either through conservation biocontrol or through inundative/augmentative releases. This paper reviews the literature on (micro-) habitat and host location by Opiinae parasitoids (Hymenoptera: Braconidae), of Tephritidae, as they are the most commonly used agents in biocontrol programs against tephritid pests [12], with some noticeable exceptions [13,14], which are only briefly covered here. 

## 2. Host Habitat/Micro-Habitat Location by Fruit Fly Parasitoids

For a parasitoid of frugivorous Tephritidae, habitat location is the location of an orchard, or a fruit tree, where a parasitoid may be able to find a suitable tephritid host at a suitable life stage in which to reproduce. At a closer scale, micro-habitat location refers to the subsequent location of a fruit or a patch of fruits. During the processes of habitat or micro-habitat location, parasitoids are known to respond to both volatile semiochemicals and visual stimuli [15]. 

### 2.1. Olfactory Stimuli

Opiinae wasps innately respond to green leaf volatiles, though this response is not restricted to the host tree and does not induce a strong attraction [16,17,18]. The generic volatiles emitted by the foliage therefore appear to play the role of an accumulation stimulus [15], concentrating the parasitoids in a preferred habitat [19]. 

In several bioassays, parasitoids have demonstrated an attraction to host fruit volatiles, without the presence of visual cues using various techniques involving field cages olfactometers, wind tunnel assays and/or electroantennography (Table 1). In a wind tunnel, [20] showed that fruit volatiles emitted by orange (*Citrus sinensis* Linnaeus) and grapefruit (*Citrus* × *paradisi* Macfadyen) attracted almost twice as many females of this parasitoid as volatiles of mango, *Mangifera indica* Linnaeus, or peach, *Prunus persica* Linnaeus. By contrast, in the same species, Eben *et al*. [21] found no difference between the positive response of the females to mango or grapefruit odors. Altuzar *et al*. [22], in wind‑tunnel bio-assays, also showed a positive response of *F. arisanus* females to synomones from guava (*Psidium guajava* Linneaus) and orange (*Citrus sinensis* Linnaeus). In addition, [23] were able to demonstrate that females of *Diachasmimorpha longicaudata* (Ashmead) are equally attracted to volatiles from extracts of decaying fruits regardless of the presence or absence of host larvae in the fruits.

Several studies on the behavior of different species of larval parasitoids have demonstrated that the presence of volatiles associated with the host increases the attractiveness of the infested fruit to the parasitoid. For *Diachasmimorpha krausii* (Fullaway), the fruit is unattractive if uninfested [24]. More than a simple additive effect due to the presence of semiochemicals from both fruit and host, this increased attractiveness is synergistic, due to the chemical modification of the plume emitted by the fermenting substrate [20,23,25]. In laboratory trials comparing different host fruits infested by tephritid larvae both suitable and unsuitable for D. krausii’s development, Ero and Clarke [26] concluded that herbivore induced non-specific host fruit wound volatiles are the major cues used by foraging females to locate their host.

### 2.2. Visual Stimuli

Visual cues are expected to be of primary importance when the female parasitoid is in close contact with the micro-habitat, when the turbulent odor plume might furnish imprecise information [18,27]. Three kinds of visual stimuli are particularly important: size, shape, and color. Laboratory bioassays using fruits or plastic ‘fruit dummies’ have demonstrated increased attractiveness with increasing host size to both *D. longicaudata *[9,20,28,29], and *F. arisanus *[29,30]. [29,30] also demonstrated a slightly stronger attractiveness of “rounded” *versus *“angulated” shapes for these parasitoids, although [9] could not determine any innate shape preference for *D. longicaudata*. Similarly, Benelli and Canale [31] were not able to demonstrate innate shape preference with naive *Psyttalia concolor *(Szépligeti)*. *Data on the response of female parasitoids to colors is more ambiguous. “Color” is mainly defined by its hue (wavelength, chromatic) and its intensity (brightness, achromatic). [32] showed that *F. arisanus *females innately respond to achromatic cues without clear hue preference. Conversely, [30] showed that the attraction and landings of females of this species foraging for hosts were stronger on yellow targets, without any significant response to achromatic cues. Studies on *D. longicaudata* [29] showed an innate preference for yellow targets, while Leyva *et al*. [20] and Segura *et al*. [9] could not determine any hue preference for females of this species. As the attraction towards yellow is also found in males, Segura *et al*. [9] concluded that this characterizes a more generalist behavior, as yellow is attractive for many insects. *Psyttalia concolor *[31], did not demonstrate any color preference, although this trait is modifiable by associative learning (see below).

**Table 1:** The response of female parasitoids (Hymenoptera: Braconidae) to host-fruit (infested and uninfested) odors. + : moderate response; ++: strong response.

Parasitoid	Host fruit	Type of equipment	Type of odor source	Presence of tephritid host larvae	Parasitoid response	Reference
*Diachasmimorpha longicaudata* (Ashmead)	Peach	Cages in a greenhouse	Rotting fruit	No	+	[23]
	Peach	Wind tunnel	Fermenting fruit	No	+	[20]
	Mango	Wind tunnel	Fermenting fruit	No	+	[20]
	Grapefruit	Wind tunnel	Fermenting fruit	No	++	[20]
	Orange	Wind tunnel	Fermenting fruit	No	++	[20]
	Grapefruit	Wind tunnel	Ripe fruit	No	+	[21]
	Mango	Wind tunnel	Ripe fruit	No	+	[21]
	Grapefruit	Wind tunnel	Ripe fruit	Yes	++	[21]
	Mango	Wind tunnel	Ripe fruit	Yes	++	[21]
	Mango	Wind tunnel, field cage	Ripe fruit	No	+	[25]
	Mango	Wind tunnel, field cage	Ripe fruit	Yes	++	[25]
	Guava	Wind tunnel	Ripe fruit	No	++	[17]
	Guava	4 way olfactometer	Rotting fruit	No	+	[33]
	Guava	4 way olfactometer	Rotting fruit	Yes	++	[33]
	Guava	Wind tunnel	Ripe fruit	No	+	[34]
*Diachasmimorpha juglandis* (Muesebeck)	Walnut	Field cage, wind tunnel	Ripe fruit	No	+	[27]
	Walnut	Field cage, wind tunnel	Ripe fruit	Yes	++	[27]
*Diachasma alloeum* (Muesebeck)	Blueberry	Olfactometer	Ripe fruit	No	+	[35]
	Blueberry	Olfactometer	Ripe fruit	Yes	++	[35]
*Diachasmimorpha krausii* (Fullaway)	Guava	Lab cage	Ripe fruit	No	-	[24]
	Guava	Lab cage	Ripe fruit	Yes	++	[24]
	Peach	Lab cage	Ripe fruit	No	-	[24]
	Peach	Lab cage	Ripe fruit	Yes	++	[24]
*Psyttalia fletcheri* (Silvestri)	Cucumber	Wind tunnel	Punctured fruit	No	+	[16]
	Pumpkin	Wind tunnel	Decaying fruit	No	++	[16]
*Psyttalia incisi *(Silvestri)	Guava	Olfactometer	Ripe fruit	No	+	[34]
	Guava	Olfactometer	Ripe fruit	Yes	++	[34]
*Fopius arisanus* (Sonan)	Guava,	Wind tunnel	Ripe fruit	No	++	[22]
	Orange	Wind tunnel	Ripe fruit	No	+	[22]
	Guava	Field cage	Sliced ripe fruit	No	+	[18]
	Orange	Field cage	Sliced ripe fruit	No	+	[18]
	Orange	Field cage	Sliced ripe fruit	Yes	++	[18]
	Zucchini	Field cage	Sliced ripe fruit	No	+	[18]
	Mango	Field cage	Sliced ripe fruit	No	+	[18,22]
	Tomato	Field cage	Sliced ripe fruit	No	+	[18]
	Indian almond	Field cage	Sliced ripe fruit	No	+	[18,22]
*Fopius carpomyie *(Silvestri)	Jujube	Olfactometer	Ripe fruit	Yes	+	[36]
*Doryctobracon areolatus *(Szépligeti)	Guava	4 way olfactometer	Rotting fruit	No	+	[33]
	Guava	4 way olfactometer	Ripe fruit	No	+	[33]
	Guava	4 way olfactometer	Rotting fruit	Yes	++	[33]
*Asobara anastrephae *(Muesebeck)(Braconidae: Alysiinae)	Guava	4 way olfactometer	Rotting fruit	Yes	++	[33]

### 2.3. Field Assessment

Various field surveys have compared the parasitisation rates of Opiinae parasitoids on host fruits of different species collected in the field. Eitam and Vargas [37,38] studying the parasitisation of *Bactrocera dorsalis *(Hendel) by *F. arisanus *identified the field preference of the parasitoid for guava, strawberry guava, *Psidium littorale* Raddi, and Indian almond, *Terminalia catappa* Linnaeus, as opposed to papaya, *Carica papaya* Linnaeus, which is probably linked to a combination of olfactory and visual stimuli. Higher parasitisation by *D. longicaudata* on *C. capitata* or *B. dorsalis* have been recorded from small fruits such as coffee, *Coffea arabica* Linnaeus [39], loquat, *Eriobotrya japonica* (Thunberg) Lindley or peach [40,41], compared to larger fruit of *Citrus* spp. [39,42]. Similarly, field studies on the Melon fly, *Bactrocera cucurbitae* (Coquillett) showed that the percentage of parasitisation by *P. fletcheri* was lower among larvae infesting cucumber (*Cucumis sativus* Linnaeus) than those infesting the wild balsam apple, *Momordica balsamina* Linnaeus [43]. Laboratory experiments later confirmed differences in the attractiveness of the fruits of different cucurbit species for the females of this parasitoid, which is probably linked with the volatile compounds emitted by different host-fruits [44]. 

Several field studies have identified an inverse correlation between parasitism rate and fruit radius. For instance, the larvae of *Rhagoletis pomonella* (Walsh) are attacked less frequently by the Opiinae braconid *Diachasmimorpha mellea* (Gahan) when they develop in apples (*Malus pumila* Miller) than in hawthorn (*Crataegus* sp.), likely because of the smaller size of hawthorn fruit [28,45]. It is frequently considered that bigger fruits make it more difficult for larval parasitoids to reach their hosts; consequently parasitoids with a longer ovipositor might exploit a wider range of fruits [46,47]. This appears somewhat conflicting with the laboratory bioassays indicated above, where wasps tend to prefer larger targets. However, bigger fruits are also susceptible to increased hosts and, even if more attractive for the wasp, may offer more protection to the larvae through a thicker pulp, thus resulting in reduced overall parasitism observed in field surveys.

Visual cues, such as coloration may be linked to level of parasitism in fruit. In a field trial, *Diachasmimorpha juglandis *(Muesebeck) preferred partly yellow walnuts rather than black walnuts, the latter which are less likely to harbour host larvae [48]. The phenological stage of the fruit may influence both the volatile emissions and visual characteristics of that fruit. Liquido [49], studying the parasitisation rate of *B. dorsalis* eggs by *F. arisanus*, showed that fruit preference was greater for fully ripe fruits of papaya, than for one-quarter to half-ripe fruits on trees. Furthermore, Eitam *et al*. [37] and Purcell *et al*. [50] showed that the fruits fallen on the ground are less attractive for *F. arisanus* than fruit on the tree. This phenomenon was also documented for four native Opiinae parasitoids of Tephritidae in Argentina [51]. The micro-habitat location by parasitoids may also be modified or even disrupted by the presence of insecticides or kaolin-clay on fruits [35].

Frequently, field surveys show that Opiinae parasitoids of frugivorous Tephritidae recognize a large variety of micro-habitats, though they exhibit clear preferences. However, such studies generally do not identify which stimuli have elicited the female response, which is important in order to understand the parasitoids attraction to fruit of a certain size, color or phenological stage. Such knowledge would assist in determining which fruit species, variety or phenological stage to release a given parasitoid species, in order to optimize the effectiveness of augmentative release.

## 3. Host Location by Fruit Fly Parasitoids

Once the micro-habitat is located, parasitoid wasps must precisely locate within the fruit the host to be parasitized. The cues used here depend on the ecology of the wasp, *i.e.*, mainly whether it parasitizes eggs or larvae [52]. They mainly differ from the cues involved in habitat location by the behavior triggered. The “attractant” stimuli used during habitat location induces a directed flight towards the source, while at a shorter distance from the host some “attractants” may be involved in host location but also “arrestants”, inducing an intense probing behavior in a small area [19]. These probings into the substrate often lead to contact with the potential host [53]. 

### 3.1. Egg Parasitoids

As eggs are concealed and immobile, all the stimuli involved here are assumed to be semiochemicals. Eggs indeed emit few semiochemicals [54], and egg parasitoids may thus rely on kairomones emitted by the host community to locate the oviposition site. Most of the studies on host location by eggl fruit fly parasitoids have been conducted on *F. arisanus*. Wang *et al*. [55] described the behavioral sequence of females foraging for host eggs after landing on a fruit. Rousse *et al*. [18] showed that female *F. arisanus* positively responds to stimuli laid by the adult hosts on visited surfaces, such as faeces. These authors also showed that females recognize a volatile kairomone coating the egg masses of various tephritid species, but did not respond to the odor of non host egg‑masses. The response to this kairomone was stronger in the case of *F. arisanus*’ preferred host, *Bactrocera zonata* (Saunders), compared to different *Ceratitis* spp., which are less preferred hosts. Although the role of this kairomone is not entirely clear, it is not a Host Marking Pheromone (HMP) [56], as *B. zonata *does not mark its oviposition sites [57]. In contrast, *Utetes canaliculatus *(Gahan), which also attacks eggs and early larval instars of its tephritid host, uses a HMP as a kairomone [58]. The use of an HMP is also evident in the egg-larval pteromalid parasitoid *Halticoptera rosae* Burks of *Rhagoletis basiola* Osten-Sacken [59]. The literature cites very few cases of host location by host kairomones in parasitoids, although it has been reported in the Scelionidae [60,61], and Trichogrammatidae [62]. Usually, egg parasitoids use non-volatile semiochemicals of eggs during the final step of host selection, *i.e.*, the host acceptation stage [15].

### 3.2. Larval Parasitoids

Larval parasitoids may rely on more cues than egg parasitoids. Some species are known to use mechanical cues to locate the vibrations of their host inside the substrate. In addition to chemoreceptors reported for example on the antennae of *P. concolor *[63] or the ovipositor of *F. arisanus *[64], it was shown that *D. longicaudata* also bears mechanical receptors on tarsi [53]. 

#### 3.2.1. Chemical Stimuli

Similar to egg parasitoids, the presence of frass laid by the host community may increase the foraging activity of larval parasitoids, as shown for *D*. *krausii* [26]. Several studies suggest that chemical cues emitted by host larva play little role in host detection. Jang *et al*. [17] showed that *D. longicaudata *does not respond to host larvae in the absence of habitat stimuli. It cannot find immobilized larvae [65], even when these hosts are unconcealed from their substrate [66]. Similarly, Ero and Clarke [26] suggest that direct larval cues are of little importance for *D. krausii *when selecting a host. Wind tunnel studies on the olfactive response of *Diachasmimorpha tryoni* (Cameron) to *Ceratitis *spp. larvae, and *P. fletcheri* to larvae of *B. cucurbitae* without any additional substrate or other cues, also showed no attractiveness [67]. However, Duan and Messing [66] showed that *D. tryoni *is capable of locating and probing immobilized host-larvae outside of their substrate, suggesting a response to chemical stimuli. Moreover, Stuhl *et al. *[68] isolated a volatile compound, ethylacetophenone, emitted by the larvae of different tephritid species. Although this compound didn’t show a long range (>1 m) of attractiveness for female *D. longicaudata*, it appears to stimulate ovipositor-insertion and oviposition. In a recent study, Segura *et al.* [9] also showed that once a female *D. longicaudata* has landed on a fruit, direct chemical cues associated with host larva activity are important for host location.

#### 3.2.2. Mechanical Stimuli

In addition to chemical stimuli directly emanating from the host, mechanical stimuli also play a role in host location by larval parasitoids. Vibrotaxis in larval parasitoids has been reviewed by Meyhöfer and Casas [69]. To date, vibrotaxis and increased probing activity related to the detection of vibrations has been unambiguously demonstrated in four Opiinae parasitoids of Tephritidae; *D. longicaudata *[65,66], *D. tryoni *[66], *Diachasma alloeum *(Muesebeck) [70], and *D. mellea* [71]. Moreover, Canale and Loni [72] linked the enhanced detection by *P. concolor* of third instar rather than second instar larvae to the higher level of vibrations produced by the former. The response to mechanical cues is of particular interest for parasitoids of fruit infesting Tephritidae, because these parasitoids are relatively generalist. Foraging for various host species feeding on numerous host fruits, they must rely on generic cues according to the principle of dietary specialization and semiochemical use [11]. 

## 4. Behavioral Plasticity

### 4.1. Learning

Another consequence of Opiinae parasitoids of frugivorous Tephritidae generally wide host range is that their foraging behavior may be influenced by associative learning [10]. Dukas and Duan [73] demonstrated that *F. arisanus*’ preferences for host fruit are modified according to the presence or absence of hosts within the fruits it previously visited. The influence of previous experience of females when foraging for an host and/or a substrate has also been emphasized in *P. concolor* [31]. Similarly, associative learning for color has been demonstrated in *D. longicaudata *[9] and *P. concolor *[31]. This learning ability has been directly linked to an increase in the wasp’s realized fecundity, reducing both foraging and handling times: such a behavioral plasticity positively affects wasp’s fitness [73]. Learning by parasitoids could for instance be manipulated in mass-rearing units in order to maximize the host searching and reproductive capability of parasitoids once released.

Besides associative learning, there is no evidence of sensitisation, *i.e.*, modification of behavior by pre-imaginal experience, in *F. arisanus* [52], or in *P. concolor. *This is of critical importance for mass‑rearing purposes, when the rearing host differs from the target pest(s). Canale and Benelli [74], and González *et al*. [75] showed that mass-rearing did not significantly affect the foraging behavior of *P. concolor *and *D. longicaudata*, respectively. Conversely, both males and females of *D. alloeum *exhibit a preference for the fruit or the host species from which they emerged [76]. Notably, *D. alloeum *is considered a specialist, with a host range restricted to two sibling species of *Rhagoletis* whereas the other cited examples are all considered generalists [12].

### 4.2. Abiotic Factors

The circadian activity of Opiinae parasitoids is related to their ecology. *Psyttalia fletcheri *exhibits a peak of host foraging activity at dawn and twilight, matching the hours at which their larval hosts leave the fruit to pupate in the soil [77]. Conversely, *F. arisanus *females forage all day long for eggs to parasitize, their availability being far less affected by the precise hour [78]. As for many insects, climatic conditions may also modulate fruit fly parasitoid foraging activity. For instance, temperature and humidity both influence the flight behavior of *F. arisanus *[78]. In addition, *D. longicaudata *[79] and *F. arisanus* [55,78] avoid flying during strong wind speed periods (>0.7 m.s^−1^), although a minimum speed of 0.4 m.s^−1^ enhances the foraging activity of the former by facilitating the dispersion of volatile semiochemicals. 

### 4.3. Physiology

Foraging behavior is also modulated by the physiological condition of the wasp, though this aspect has been less documented for Opiinae parasitoids. In *F. arisanus*, foraging activity is correlated with egg load [78]. As Opiinae parasitoids are synovogenic species, their potential fecundity should be linked with age and nutritional status. Under laboratory conditions, the maximal potential fecundity of *D. longicaudata *[80] and *F. arisanus *[81] is reached about 10 days after emergence. It was moreover confirmed in *Opius hirtus *Fischer, *D. longicaudata *and *Utetes anastrephae *(Viereck) that a high quality adult diet positively influences egg load and/or egg maturation rate [80]. Furthermore, oosorption has been shown in *F. arisanus *[64], commencing four days after the absence of hosts [82]. On the other hand, no clear trade-off between host and food foraging could be demonstrated for *F. arisanus* starved females [78].

## 5. Pupal Parasitoids

The pupal stage of tephritids is also parasitized by numerous parasitoids in the field, mostly Chalcidoidea and Evanioidea. Many are generalists, and several hyperparasitize Opiinae. Resultingly, they are of lesser interest for biocontrol purposes. The most promising exception is probably the endoparasitoid *Coptera haywardi *(Oglobin) (Hymenoptera: Diapriidae), reported in Latin and Central America as far north as Mexico [83]. Unlike other pupal parasitoids of cyclorrhaphous dipterans, it is rather specialized, its host range being restricted to three genera of Tephritidae, namely *Ceratitis Anastrepha*, and *Toxotrypana *[13]. As *C. haywardi* is a specialist, it is thought that semiochemicals emitted by the host may be involved in its host location process. The ability of *C. haywardi* to forage for host pupae in the soil was assessed [84,85], showing for example that it locates deeper buried pupae than do some more generalist parasitoids. Additionally, inter-specific competition trials showed that *C. haywardi *efficiently discriminates and avoids pupae which have been parasitized by *D. longicaudata *[86], making it a promising candidate for its combined use with larval parasitoids. 

## 6. Conclusion and Perspectives

A detailed knowledge of the different steps of host habitat and host location behavior of fruit fly parasitoids may be very useful for the planning of both inundative/augmentative releases of mass‑reared parasitoids and inoculative releases for classical biocontrol. Such release sites could be selected not only based on the abundance of favorable hosts at a suitable stage, but also on the presence of plants known to induce the highest attractive response for the parasitoid females. For instance, carefully designed mixed fruit orchards could provide a combination of attractive stimuli for tephritid parasitoids at a given period, or alternatively a succession of attractive stimuli from different host-fruits at different periods of the year. Associative learning could be used to optimize pre-release conditions of mass-reared parasitoids by exposing them to selected stimuli they are likely to meet in the biotopes chosen for releases. Finally the influence of abiotic factors should be taken into account to optimize the timing and conditions of releases. 

Additional research is still required to improve our knowledge of the chemical compounds emitted by infested fruits that induce the highest response of females of both egg and larval parasitoids [12,18]. Similarly, the chemical composition of kairomones whether deposited by the female fruit fly, on the egg or near the oviposition puncture, should be further investigated, as well as volatiles produced by fruit fly larvae or those resulting from their activity. Such parasitoid attractants, even though their radius of action may be limited, could possibly be useful for augmentative releases; by helping maintain fruit fly parasitoidsin suitable target areas.

Some physiological and biochemical factors which modulate the initiation of female parasitoid oviposition could be manipulated to maximize a mass-reared parasitoids impact upon release. For example, different sources of carbohydrates might be evaluated as pre-release food to maximize parasitoid fitness. Additionally, understanding how the insects process the host location information centrally, is a largely unexplored research field which might further enlarge our understanding of parasitoid behavior.
